# Functional analyses of ATM, ATR and Fanconi anemia proteins in lung carcinoma: ATM, ATR and FA in lung carcinoma

**DOI:** 10.1186/s12885-015-1649-3

**Published:** 2015-10-05

**Authors:** Jan H. Beumer, Katherine Y. Fu, Bean N. Anyang, Jill M. Siegfried, Christopher J. Bakkenist

**Affiliations:** 1 Department of Pharmaceutical Sciences, University of Pittsburgh School of Pharmacy, Pittsburgh, PA USA; 2 Molecular Therapeutics Drug Discovery Program, University of Pittsburgh Cancer Institute, Pittsburgh, PA USA; 3 Department of Radiation Oncology, University of Pittsburgh School of Medicine, Pittsburgh, PA USA; 4 Department of Pharmacology, Masonic Cancer Center, University of Minnesota Medical School, Minneapolis, MN USA; 5 Department of Pharmacology and Chemical Biology, University of Pittsburgh School of Medicine, Pittsburgh, PA USA; 6 Hillman Cancer Center, Research Pavilion, Suite 2.6, 5117 Centre Avenue, Pittsburgh, PA 15213-1863 USA

**Keywords:** ATM, ATR, Fanconi anemia, Lung carcinoma

## Abstract

**Background:**

ATM and ATR are kinases implicated in a myriad of DNA-damage responses. ATM kinase inhibition radiosensitizes cells and selectively kills cells with Fanconi anemia (FA) gene mutations. ATR kinase inhibition sensitizes cells to agents that induce replication stress and selectively kills cells with *ATM* and *TP53* mutations. *ATM* mutations and *FANCF* promoter-methylation are reported in lung carcinomas.

**Methods:**

We undertook functional analyses of ATM, ATR, Chk1 and FA proteins in lung cancer cell lines. We included Calu6 that is reported to be FANCL-deficient. In addition, the cancer genome atlas (TCGA) database was interrogated for alterations in: 1) *ATM, MRE11A, RAD50* and *NBN*; 2) *ATR, ATRIP* and *TOPBP1*; and 3) 15 FA genes.

**Results:**

No defects in ATM, ATR or Chk1 kinase activation, or FANCD2 monoubiquitination were identified in the lung cancer cell lines examined, including Calu6, and major alterations in these pathways were not identified in the TCGA database. Cell lines were radiosensitized by ATM kinase inhibitor KU60019, but no cell killing by ATM kinase inhibitor alone was observed. While no synergy between gemcitabine or carboplatin and ATR kinase inhibitor ETP-46464 was observed, synergy between gemcitabine and Chk1 kinase inhibitor UCN-01 was observed in 54 T, 201 T and H460, and synergy between carboplatin and Chk1 kinase inhibitor was identified in 201 T and 239 T. No interactions between ATM, ATR and FA activation were observed by either ATM or ATR kinase inhibition in the lung cancer cell lines.

**Conclusions:**

Analyses of ATM serine 1981 and Chk1 serine 345 phosphorylation, and FANCD2 monoubiquitination revealed that ATM and ATR kinase activation and FA pathway signaling are intact in the lung cancer cell lines examined. As such, these posttranslational modifications may have utility as biomarkers for the integrity of DNA damage signaling pathways in lung cancer. Different sensitization profiles between gemcitabine and carboplatin and ATR kinase inhibitor ETP-46464 and Chk1 kinase inhibitor UCN-01 were observed and this should be considered in the rationale for Phase I clinical trial design with ATR kinase inhibitors.

**Electronic supplementary material:**

The online version of this article (doi:10.1186/s12885-015-1649-3) contains supplementary material, which is available to authorized users.

## Background

Ataxia telangiectasia mutated (ATM) and ATM and Rad3-related (ATR) are kinases implicated in a myriad of DNA damage responses [1]. Somatic mutations in *ATM* were identified previously in 14 of 188 lung adenocarcinomas (7 %) [2]. While the functional significance of the *ATM* mutations identified has not been determined, ATM polymorphisms are known to affect lung cancer risk [3]. Further, since ataxia telangiectasia individuals with mutations in the *ATM* gene are extremely radiosensitive, ATM kinase inhibition is expected to increase the efficacy of radiotherapy [4, 5]. Consistent with this expectation, three small-molecule, selective ATM kinase inhibitors radiosensitize cells *in vitro* [6–9]. Thus, up to 7 % of lung adenocarcinomas that have acquired somatic mutations that inactivate ATM may respond extremely well to radiotherapy, while lung cancers that express functional ATM are anticipated to be radiosensitized by ATM kinase inhibitors.

ATM kinase inhibitors also kill cell lines with mutations in genes that cause Fanconi anemia (FA), a multigenic disorder characterized by extreme sensitivity to interstrand crosslinks (ICLs), with greater efficacy than complemented control cell lines [10, 11]. Inactivation of the FA pathway through promotor methylation of *FANCF* was identified previously in 22 of 158 non-small-cell lung carcinomas (NSCLCs) (14 %) [12]. Thus, up to 14 % of NSCLCs may respond to single agent therapy with an ATM kinase inhibitor.

In contrast to ATM, ATR is an essential protein in mice and ATR disruption by genetic means kills human cells *in vitro* [13]. However, Seckel syndrome individuals have a mutation in a splice site that results in the expression of just 10 % of the typical levels of ATR protein, which allows them to survive [14]. Since cells derived from Seckel syndrome individuals are extremely sensitive to mitomycin C (MMC) and ultraviolet radiation, ATR kinase inhibition is expected to increase the efficacy of chemotherapeutics that induce replication stress. Consistent with this expectation, three small-molecule selective ATR kinase inhibitors sensitize cells to agents that induce replication stress *in vitro* [15–17]. ATR kinase inhibitors also kill cell lines with mutations in either *ATM* or *TP53* with greater efficacy than complemented control cell lines. Thus, up to 7 % of lung adenocarcinomas that have acquired somatic mutations that inactivate ATM may respond to single agent therapy with an ATR kinase inhibitor.

Here we sought to elucidate whether the ATM, FA and ATR pathways interact with each other and whether the ATM, FA and ATR pathways may be new diagnostic and therapeutic biomarkers for lung cancer.

## Materials and methods

### Ethics

No research involving human subjects or human material is described in this manuscript.

### Cell culture

54 T, 201 T and 239 T are NSCLC cell lines generated from primary patient tissues at the University of Pittsburgh [18]. H460 and Calu6 were purchased from American Type Culture Collection (ATCC). Cells were treated with 0.2 μM MMC, 0.1 μM gemcitabine or carboplatin (Sigma Aldrich, St. Louis, MO). ATM kinase inhibitors KU55933 [6] and KU60019 [7] (AstraZeneca, Macclesfield, UK) were used at final concentrations of 10 μM and 1 μM, respectively. ATR kinase inhibitor ETP-46464 was used at a final concentration of 10 μM [15]. ETP46464 was synthesized at the Medicinal Chemistry Shared Resource of the Ohio State University Comprehensive Cancer Center (Columbus, OH). Cells were γ-irradiated in a Shepherd Mark I Model 68 [^137^Cs] irradiator (J.L. Shepherd & Associates, San Fernando, CA) at a dose rate of 71.1 Rad/min.

### Immunoblotting

Rabbit monoclonal anti-ATM 1981S-P (EP1890Y, Epitomics, Burlingame, CA), mouse monoclonal anti-ATM antisera (MAT3-4G10/8, Sigma-Aldrich, St. Louis, MO), anti-p53 15S-P (9284, Cell Signaling Technology, Danvers, MA), anti-p53 (sc6243-G, Santa Cruz Biotechnology, Santa Cruz, CA), anti-Chk1 S345-P (2348S, Cell Signaling), and anti-Chk1 2G1D5 (2360, Cell Signaling) were used. Whole cell extracts were prepared in: 50 mM Tris-HCl pH 7.5, 150 mM NaCl, 50 mM NaF, 1 % Tween-20, 0.5 % NP40 and 1 × protease inhibitor mixture (Roche Applied Science, Indianapolis, IN).

### Clonogenic survival assays

Cells were prepared in suspension and treated with KU60019 and increasing doses of ionizing radiation (IR). Drug treatments were removed 17 h post-IR. After 10 days, colonies were stained with crystal violet stain. All experiments were performed in triplicate.

### Proliferation assays

MTT Assay (Trevigen, Gaithersburg, MD) was used to measure cell proliferation. Drug combinations were evaluated using CalcuSyn (BIOSOFT, Ferguson, MO) software based on the multiple drug effect equation of Chou-Talalay. Experimental values were imputed into Calcusyn to calculate IC50 and a combination index (CI, a quantitative measure of the synergy (CI < 1), additivity (CI = 1), or antagonism (CI > 1) between drugs).

Log-transformed CI’s are plotted against growth inhibition/effective dose (ED) with corresponding 95 % confidence intervals. Synergism is indicated when the 95 % CI falls below the x-axis (log CI = 0; CI = 1), whereas antagonism is indicated when the 95 % CI falls above the x-axis, at each respective region of the effective dose.

### TCGA analyses

Analyses were undertaken using the cBio Cancer Genomics Portal at Memorial Sloane Kettering Cancer Center [19]. At the time of writing, the following analyses had been completed on this dataset: sequenced, 183; array-comparative genomic hybridization (aCGH), 179; tumor RNA-seq, 178; tumor mRNA microarray, 154; tumor miRNA, 317; and methylation, 133.

## Results

### Functional analyses of ATM kinase activity in lung cancer cell lines

**Fig. 1:**
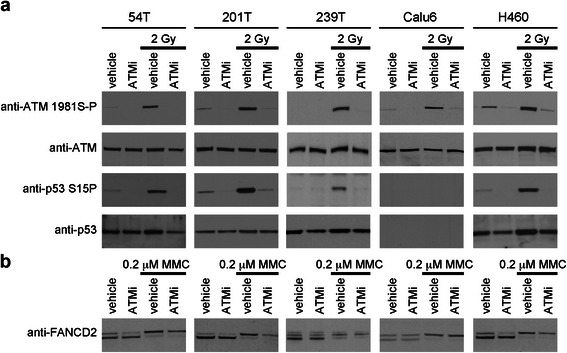
**a**: The kinase activity of ATM was increased in lung cancer cell lines exposed to IR. Exponentially dividing lung cancer cell lines were exposed to ATM kinase inhibitor KU60019 (ATMi) for 15 min. Cells were exposed to 2 Gy IR. Whole cell extracts were prepared at 1 h post-IR, resolved and immunoblotted as indicated. **b**: FANCD2 was covalently modified in lung cancer cell lines exposed to an agent that induces ICLs. Exponentially dividing lung cancer cell lines were exposed to 100 nM MMC and KU60019 for 18 h. Whole cell extracts were prepared and immunoblotted as indicated. ICL-induced FANCD2 mobility shift (arrow) is seen in all the lung cancer cell lines examined and this shift is not inhibited by ATM kinase inhibitor

ATM serine 1981 phosphorylation is associated with ATM kinase activity, and alterations in *ATM, MRE11A, RAD50* and *NBN* may disrupt this biomarker for functionality of ATM kinase activation mechanisms [20]. ATM kinase-dependent, ATM serine 1981 phosphorylation was induced by IR in all cell lines (Fig. 1a). Mechanisms of ATM kinase activation as determined by ATM serine 1981phosphorylation are thus intact in these cell lines.

Calu6 is being sequenced in the Catalogue of Somatic Mutations in Cancer (COSMIC) Cell Lines Project at the Sanger Center, Cambridge, UK and has a homozygous missense point mutation (R196*) in *TP53*. Of the other cell lines, H460 is a large cell carcinoma with *wild-type TP53*, 201 T is a lung adenocarcinoma with *wild-type TP53*, and 54 T and 239 T are lung squamous cell carcinomas with *wild-type TP53*. We selected lung cancer cell lines with *wild-type TP53* for this study as we sought to identify the somatic mutations that compromised ATM and ATR kinase-dependent signaling to p53. ATM kinase-dependent IR-induced p53 serine 15 phosphorylation was seen in 54 T, 201 T, 239 T and H460 (Fig. 1a).

### Functional analyses of the FA pathway in lung cancer cell lines

A complex of FANCA, FANCB, FANCC, FANCE, FANCF, FANCG, FANCL*,* and FANCM comprise the FA core complex that monoubiquitinates FANCD2 and FANCI following DNA damage [21]. Monoubiquitinated FANCD2 can be resolved from unmodified FANCD2 in SDS-PAGE and this “bandshift” is a biomarker for the functionality of the FA core complex. ICL-induced monoubiquitinated FANCD2 was observed in all cell lines (Fig. 1b). FA core functionality is thus intact in all cell lines. This data conflicts with a previous report that ICL-induced monoubiquitinated FANCD2 and FANCL protein were not detected in Calu6 [12]. We submitted our Calu6 to ATCC for authentication and 100 % of the markers examined were coincident between our Calu6 and those at the ATCC. We purchased new Calu6 cells from the ATCC. ICL-induced monoubiquitinated FANCD2 was observed in the new Calu6. We conclude that FA core functionality is intact in Calu6.

### Analyses of lung cancer cell line killing by ATM kinase inhibitors and IR

ATM kinase inhibitors radiosensitize cells *in vitro* [6–9]. ATM kinase inhibitors also kill cell lines containing mutations in FA genes [11]. While ATM kinase inhibitor radiosensitized 201 T, 239 T, Calu6 and H460, ATM kinase inhibitor did not kill lung these lung cancer cell lines in the absence of exogenous DNA damage (Additional file 1: Figure S1). 54 T is not included in these data since these cells did not form colonies.

### Functional analyses of ATR kinase activity in lung cancer cell lines

While ATM kinase activity is increased in response to DSBs, ATR kinase activity is increased by replication stress. However, from a therapeutic perspective these two kinases interact as ATM kinase inhibition causes DSBs to accumulate in cells and these activate ATR kinase as they are repaired by homologous recombination repair (HRR). Further, ATR kinase inhibition causes stalled replication forks to collapse and these activate ATM kinase when they are cleaved by endonucleases.

**Fig. 2:**
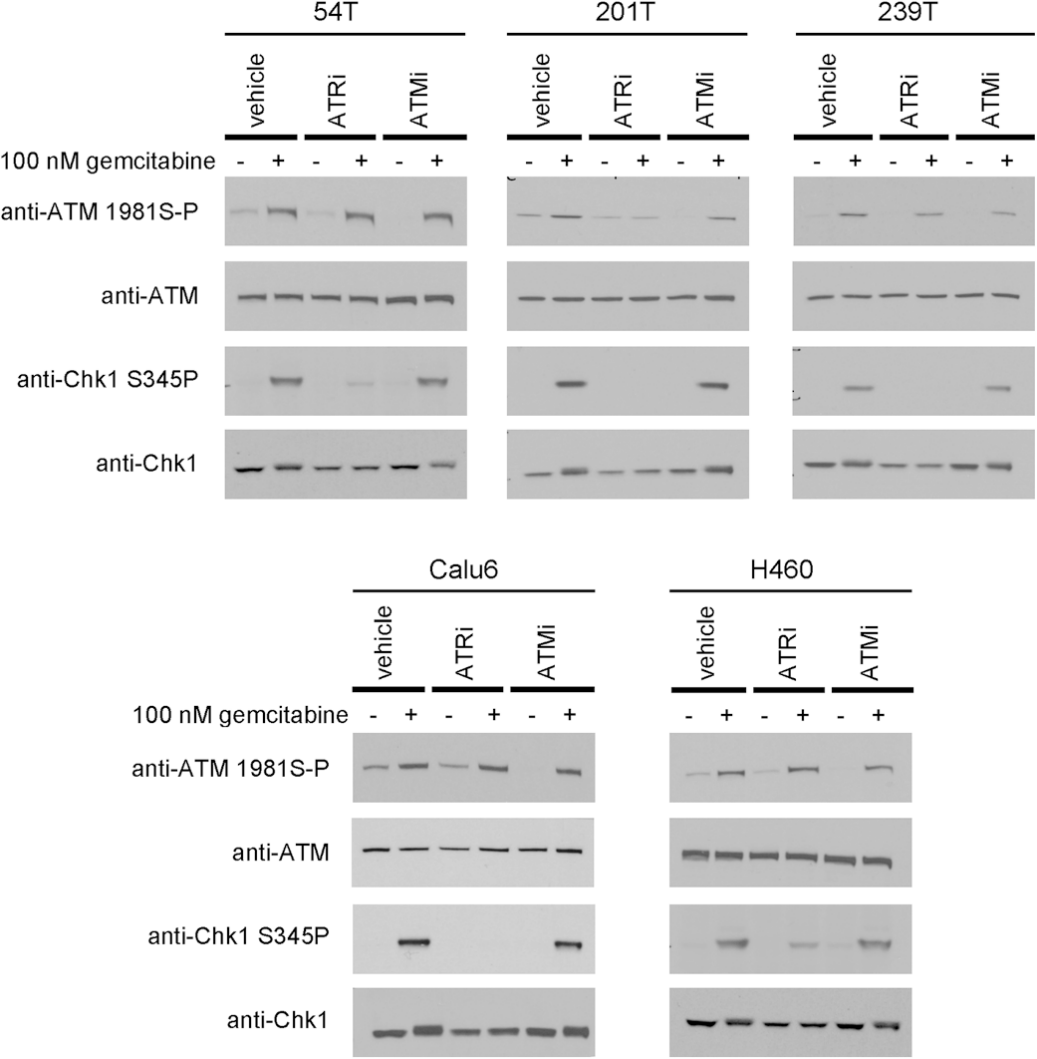
ATR kinase-dependent, Chk1 serine-345 phosphorylation was induced by gemcitabine in all cell lines. Exponentially dividing lung cancer cell lines were exposed to gemcitabine and ATR kinase inhibitor ETP-46464 (ATRi) or ATM kinase inhibitor KU55933 (ATMi) for 4 h. Whole cell extracts were prepared, resolved and immunoblotted as indicated

An ATR kinase-dependent phosphorylation on Chk1 serine-345 is required for Chkl activation, and alterations in *ATR, ATRIP* and *TOPBP1* may disrupt this biomarker for functionality of ATR kinase activation mechanisms [22]. ATR kinase-dependent Chk1 serine-345 phosphorylation was induced by gemcitabine in all cell lines (Fig. 2). Mechanisms of ATR kinase activation are thus intact in these cell lines. While ATR kinase inhibitor disrupts gemcitabine-induced Chk1 serine 345 phosphorylation, gemcitabine-induced ATM serine 1981 phosphorylation is not disrupted in 54 T, 239 T, Calu6 or H460 by either ATM or ATR kinase inhibitor (Fig. 2). ATM serine 1981 phosphorylation is ATM kinase-dependent in cells exposed to agents that induce DSBs [20]. However, ATM serine 1981 phosphorylation has been shown to require ATR in cells exposed to agents that induce stalled replication forks [23]. It is possible that gemcitabine-induced ATM serine 1981 phosphorylation is both ATM and ATR kinase-dependent in these lung cancer cell lines and that inhibition of either kinase is insufficient to significantly reduce the phosphorylation. It is also possible that ATM is phosphorylated by a different class of kinase and a recent report that IKKβ phosphorylates ATM on serine 1981 in cells exposed to alkylating agents is provocative [24].

### Analyses of lung cancer cell line killing by ATR kinase inhibitors and gemcitabine

**Fig. 3:**
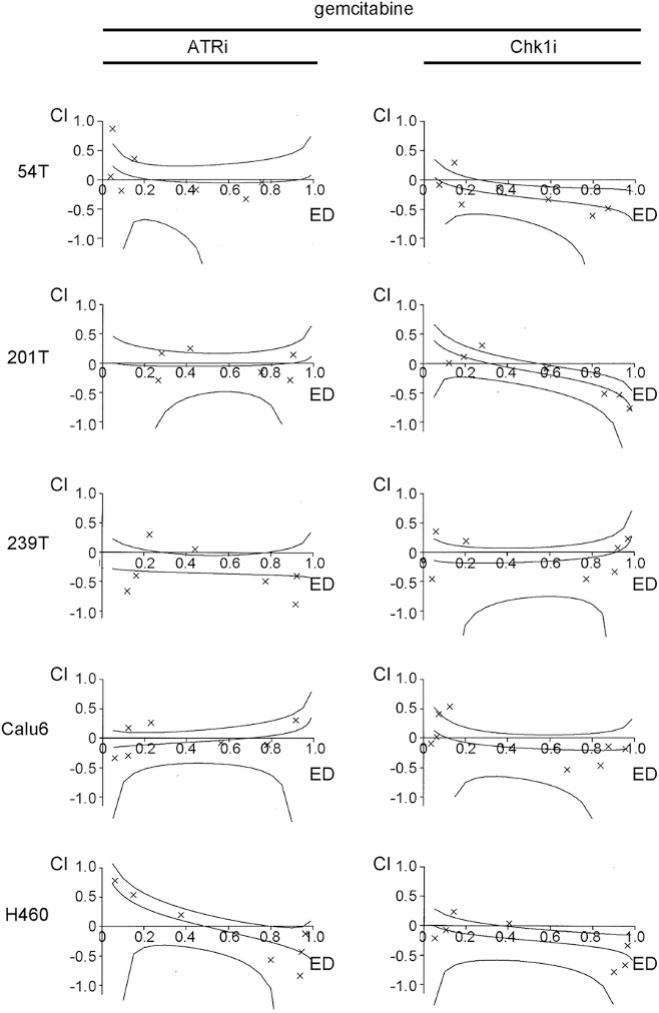
Synergy in cell killing was seen between gemcitabine and Chk1 kinase inhibitor (UCN-01) in 54 T, 201 T, at the higher response range, and H460 (Fig. 6, Table 1). However, no synergy was seen between gemcitabine and ATR kinase inhibitor (ETP-46464). Exponentially dividing lung cancer cell lines were treated with increasing doses of gemcitabine, ATR kinase inhibitor ETP-46464 and Chk1 kinase inhibitor UCN-01 for 48 h and MTT reagent was then added. Calcusyn was used to calculate a combination index (CI), a quantitative measure of the synergy (CI < 1), additivity (CI =1), and antagonism (CI > 1) between drugs. Log-transformed combination indices (CI) are plotted against growth inhibition/effective dose (ED) with corresponding 95 % confidence interval for a representative experiment. Representative examples from at least 3 experiments are shown (mean of 3 replicates)

We were interested to investigate cell killing by ATR kinase inhibitor. We employed gemcitabine as a DNA damaging agent to induce stalled replication forks that are not associated with ICLs. This was because we were initially concerned that ICLs would accumulate in FA-deficient lung cancer cell lines. We employed both ATR and Chk1 kinase inhibitors since these kinases are in the same signaling pathway. Synergy in cell killing was seen between gemcitabine and Chk1 kinase inhibitor (UCN-01) in 54 T, 201 T and H460 at the higher response range (Fig. 3, Additional file 2: Table S1). Chk1 kinase inhibition was recently reported to increase sensitivity to gemcitabine in two p53 mutant NSCLC cell lines with either high (H1299) or low (H1993) Chk1 [25]. However, no synergy was seen between gemcitabine and ATR kinase inhibitor. Thus, ATR kinase inhibitor ETP-46464 and Chk1 inhibitor UCN-01 do not phenocopy each other in combination with gemcitabine.

### Analyses of lung cancer cell line killing by ATM kinase inhibitors and gemcitabine

**Fig. 4:**
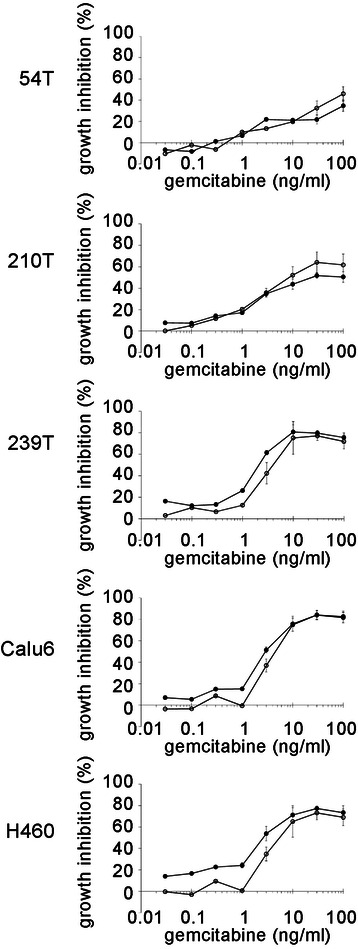
No synergy was seen between gemcitabine and ATM kinase inhibitor. Exponentially dividing lung cancer cell lines were treated with increasing doses of gemcitabine and a fixed concentration of ATM kinase inhibitor KU55933 for 48 h and MTT reagent was then added. Representative examples from at least 3 replicate experiments are shown (mean, SD of 4 replicates)

We were also interested in how cell killing by gemcitabine would be affected by ATM kinase inhibition. We reasoned that gemcitabine might induce DSBs in lung cancer cell lines due to acquired mutations that disrupt mechanisms that protect stalled replication forks. If this were the case then increased cell killing might be seen with gemcitabine and ATM kinase inhibitor but not gemcitabine and ATR kinase inhibitor. However, gemcitabine was not potentiated by ATM kinase inhibition (Fig. 4, Additional file 2: Table S2). Thus, the lesions induced by gemcitabine and ATM kinase inhibition do not interact in the lung cancer cell lines examined.

### Analyses of lung cancer cell line killing by ATR kinase inhibitors and carboplatin

**Fig. 5:**
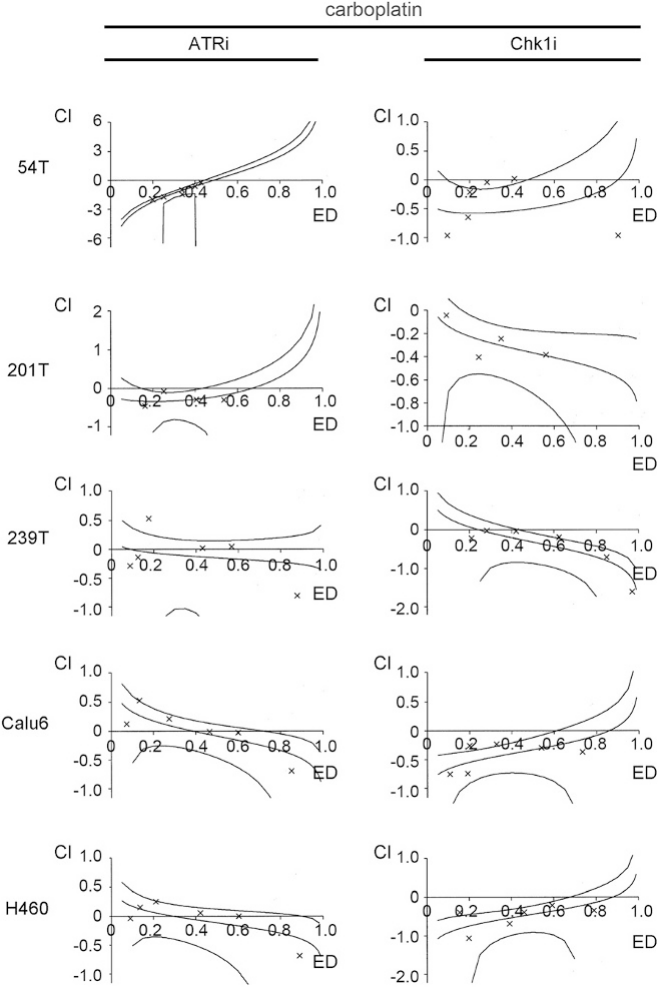
Synergy in cell killing was seen between carboplatin and Chk1 kinase inhibitor (UCN-01) in 239 T, at the higher response range, and 201 T. However, synergy was only seen in 54 T between carboplatin and ATR kinase inhibitor (ETP-46464) at the lower dose range. Exponentially dividing lung cancer cell lines were treated with increasing doses of carboplatin, ATR kinase inhibitor ETP-46464 and Chk1 kinase inhibitor UCN-01 for 48 h and MTT reagent was then added. Calcusyn was used to calculate a combination index (CI), a quantitative measure of the synergy (CI < 1), additivity (CI =1), and antagonism (CI > 1) between drugs. Log-transformed combination indices (CI) are plotted against growth inhibition/effective dose (ED) with corresponding 95 % confidence interval for a representative experiment. Representative examples from at least 3 experiments are shown (mean of 3 replicates)

Since the FA pathway was intact in all the cell lines examined we used carboplatin to induce stalled replication forks at ICLs. Synergy in cell killing was seen between carboplatin and Chk1 inhibitor in 239 T at the higher dose range and in 201 T (Fig. 5, Additional file 2: Table S3). Thus, while no synergy between gemcitabine or carboplatin and ATR inhibition was observed in the lung cancer cell lines used here, synergy between gemcitabine and Chk1 inhibition was observed in 54 T, 201 T and H460, and synergy between carboplatin and Chk1 inhibition was identified in 201 T and 239 T. This contrasts with a recent report that shows synergy between carboplatin and ATR kinase inhibitor ETP-46464 in ovarian cancer cell lines [26] and data that documents synergy between carboplatin and another ATR kinase inhibitor [17]. Chk1 kinase inhibition, but not ATR kinase inhibition, blocks a mechanism(s) that is essential for survival in certain lung cancer cell lines treated with either gemcitabine or carboplatin.

### Analyses of ATM, FA, and ATR alterations in 212 lung squamous cell carcinomas (TCGA)

**Fig. 6:**
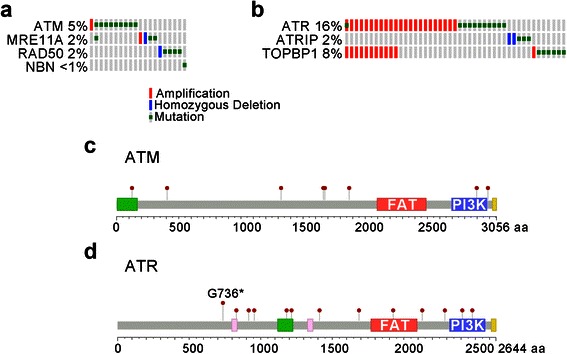
**a**. Alterations in *ATM, MRE11A, RAD50* and *NBN* were identified in 212 lung squamous cell carcinomas (TCGA). Amplification, homozygous deletion and mutation are shown. **b**. Alterations in *ATR, ATRIP* and *TOPBP1*. **c**. Mutations in ATM. **d**. Mutations in ATR. G736* occurs in two independent carcinomas

Mechanisms of ATM and ATR kinase activation and FA core functionality are intact in the cell lines examined. To extend these findings we interrogated the publically available database of 212 lung squamous cell carcinomas in the TCGA to determine the incidence of alterations that are predicted to compromise ATM and ATR kinase activation and the FA pathway of ICL repair. ATM kinase activation following low doses of IR requires the MRE11A, RAD50 and NBN complex [27, 28]. The TCGA database contains 9 missense point mutations in *ATM*, 3 in *MRE11A*, 4 in *RAD50*, and 1 in *NBN* (Fig. 6a, c). *ATM* and *MRE11A* are each amplified in a single carcinoma. *MRE11A* and *RAD50* homozygous deletion are each identified in carcinomas. This is noteworthy because *MRE11A* and *RAD50* are essential genes in mice [29, 30]. Together these 4 genes that are required for ATM kinase activation are altered in 20/212 (9 %) of lung squamous cell carcinomas (TCGA).

ATR activation requires ATRIP and TOPBP1 [31, 32]. The TCGA database contains 13 missense point mutations in *ATR*, 3 in *ATRIP*, and 7 in *TOPBP1* (Fig. 6b, d). *ATR* (located on human chromosome 3q22-q24) and *TOPBP1* (located on human chromosome 3q22.1) are co-amplified in 11 carcinomas. *TOPBP1* and *ATR* are amplified independently in 1 and 12 carcinomas, respectively. *ATRIP* homozygous deletion is identified in two carcinomas. This is noteworthy because homozygous loss-of-function of ATRIP is not compatible with mammalian cell viability. Together these 3 genes that are required for ATR kinase activation are altered in 45/212 (21 %) of lung squamous cell carcinomas (TCGA). The striking conclusion from these analyses is that while ATM is mutated in a subset of lung cancers, ATR is amplified in subset of lung cancers.

At least 15 gene products constitute the FA pathway that resolves ICLs encountered by DNA replication forks. Together the 15 FA genes are altered by missense point mutation, amplification or homozygous deletion in 72/212 (34 %) of lung squamous cell carcinomas in the publically available TCGA database (Additional file 3: Figure S1). The only FA proteins in which missense point mutations are not identified were *FANCD2* and *RAD51C*. The data are summarized as follows: 6 missense point mutations in *FANCA*; 4 in *FANCB*; 2 in *FANCC*; 1 in *FANCE*; 3 in *FANCF*; 5 in *FANCG*; 1 in *FANCL*; 10 in *FANCM*; 1 in *FANCI*; 12 in *BRCA2*; 6 in *BRIP1*; 6 in *PALB2 (FANCN)*; and 14 in *SLX4 (FANCP)*. Amplifications of 8 FA genes are identified across 22 carcinomas; 2 of the carcinomas contain amplification in two FA genes (*FANCG* with *FANCI* and *BRIP1* with *RAD51C*). *FANCG*, *PALB2* and *SLX4* homozygous deletions are identified in single carcinomas while *FANCM* homozygous deletions are identified in 2 carcinomas. This is noteworthy because while *Fancg, Fancm* and *Slx4* are not essential for viability [33–35], *Palb2* is embryonically essential in mice [36].

Since inactivation of the FA pathway through methylation of the *FANCF* promoter was identified previously in 22 of 158 NSCLCs (14 %), we also examined mRNA expression levels for the FA genes in lung squamous cell carcinomas in the publically available TCGA database. Together the 15 FA genes are altered by missense point mutation, amplification, homozygous deletion, up-regulation (RNA), and down-regulation (RNA) in 102/212 (48 %) of lung squamous cell carcinomas (TCGA) (Additional file 3: Figure S1). In line with the reported inactivation of the FA pathway through methylation of the *FANCF* promoter [12], down-regulation of *FANCF* RNA is identified in 5/212 lung squamous cell carcinomas in the publically available TCGA database.

## Discussion

Somatic mutations in ATM have been identified previously in 14 of 188 lung adenocarcinomas (7 %) (2). ATM kinase activation and signaling were normal in the lung cancer cell lines examined here. Missense point mutations of *ATM* in 9/212 lung squamous cell carcinomas (4 %) are present in the TCGA database. These heterozygous mutations span the gene and aside from one mutation in the phosphatidylinositol 3-kinase domain (G2897S) none are judged likely to have a significant impact on kinase activity or expression. Missense point mutations in an extended analysis of *ATM, MRE11A, RAD50* and *NBN* are present in 16/212 lung squamous cell carcinomas (7 %) are present in the TCGA database. None of the missense point mutations in *MRE11A, RAD50* and *NBN* are judged likely to have a significant impact on ATM kinase activity or expression. Thus, our analysis does not suggest that a significant number of lung squamous cell carcinomas will be radiosensitive as a result of acquired missense point mutations that affect ATM kinase activation.

Inactivation of the FA pathway through promotor methylation of *FANCF* was also identified previously in 22 of 158 non-small-cell lung carcinomas (NSCLCs) (14 %) [12]. FA pathway activation was normal in the lung cancer cell lines examined here. Down-regulation of *FANCF* mRNA is present in only 5/212 lung squamous cell carcinomas (2 %) in the publically available TCGA database. Together missense point mutation, amplification or homozygous deletion in the 15 FA genes are present in 72/212 lung squamous cell carcinomas (34 %) in the TCGA database. Of the 52 missense point mutations in the 15 FA genes, 4 generate stop codons. None of the remaining missense point mutations are predicted to have a “high” impact on protein function. In summation, our analysis does not suggest that a significant number of lung squamous cell carcinomas will be sensitive to ICLs as a result of acquired missense point mutations that affect FA gene products.

Carcinomas with homozygous deletions in either *MRE11A, RAD50, ATRIP* or *PALB2,* 4 genes that are essential for mammalian cell viability, are present in the publically available TCGA database. These data were derived using GISTIC, a copy-number analysis algorithm and are defined by “-2,” deep loss, possible homozygous deletion. The simplest biological explanation for these observations is that the carcinomas are heterogenous and contain two populations of cells that have lost different alleles of the gene. It is unlikely transformed cells can survive without MRE11A, RAD50, ATRIP or PALB2 although significantly reduced levels may be tolerated, as evidenced by the ATR expression in Seckel syndrome and hypomorphic MRE11A and RAD50 mutations in ATLD and NBS-like disorder patients, respectively [37, 38].

*ATR, ATRIP* and *TOPBP1* are altered in 45/212 lung squamous cell carcinomas (21 %) in the TCGA database. One frame-shift mutation, one point mutation G736* (in two independent carcinomas), and one point mutation D1687H in *ATR* are likely to reduce ATR activity. In contrast, the co-amplification of *ATR* (located on human chromosome 3q22-q24) and *TOPBP1* (located on human chromosome 3q22.1) in 11 carcinomas may increase ATR activity. These data were derived using GISTIC, a copy-number analysis algorithm and are defined by “+2,” high-level amplification, possible amplification and as such are subject to similar error as the data describing homozygous deletion. However, the co-amplification of *ATR* and *TOPBP1* may serve as independent experimental validations of amplification of chromosome 3q22-q24. Certainly, amplification of *ATR* and *TOPBP1* has been separated in other carcinomas and the trend in the TCGA database is towards inactivation of ATM kinase signaling and increased ATR kinase signaling (Fig. 6).

Oncogene-induced replication stress activates the ATR pathway in many neoplasias including lung [39, 40]. Therefore, the ATR kinase signaling may be a tumor suppressor mechanism. However, alterations that compromise ATR kinase signaling may be selected against since transformed cells may have an increased dependency on the ATR pathway, analogous to oncogene addiction, to continue to replicate and divide in the presence of replication stress. However, and in contrast to expectations, no synergy between gemcitabine or carboplatin and ATR kinase inhibitor ETP-46464 was observed. In contrast synergy between gemcitabine and Chk1 inhibition was observed in 54 T, 201 T and H460, and synergy between carboplatin and Chk1 inhibition was identified in 201 T and 239 T. As such, Chk1 kinase inhibition, but not ATR kinase inhibition, blocks a mechanism(s) that is essential for survival in some lung cancer cell lines treated with either gemcitabine or carboplatin. Different sensitization profiles between ATR kinase and Chk1 kinase inhibitors have been recently published in ovarian cancer cell lines using ATR kinase inhibitor VE-821 [41], and lung cancer cell lines using ATR kinase inhibitor VE-822 and Chk1 kinase inhibitor AZD7762 [42]. Our data may be attributed to a Chk1 kinase-dependent mechanism that is ATR kinase-independent. Alternatively, Chk1 kinase inhibition may be dominant inhibitory over a survival pathway where ATR kinase inhibition is not, perhaps because an alternate mechanism can be recruited in the absence of ATR kinase signaling. Finally, ETP-46464 may inhibit a signaling pathway, in addition to that initiated by ATR kinase, that protects cells against the cytotoxic effects of ATR and Chk1 kinase inhibition. In any event, our data show that ATR kinase inhibition with ETP-46464 does not phenocopy Chk1 kinase inhibition with UCN-01 and as a consequence, ATR and Chk1 inhibitors may different sensitization profiles and this should be considered in the rationale for Phase I clinical trial design with ATR kinase inhibitors.

## Conclusions

Analyses of ATM serine 1981 and Chk1 serine 345 phosphorylation, and FANCD2 monoubiquitination revealed that ATM and ATR kinase activation and FA pathway signaling are intact in the lung cancer cell lines examined. As such, these posttranslational modifications may have utility as therapeutic biomarkers for the integrity of DNA damage signaling pathways in lung cancer. Different sensitization profiles between gemcitabine and carboplatin and ATR kinase inhibitor ETP-46464 and Chk1 kinase inhibitor UCN-01 were observed and this should be considered in the rationale for Phase I clinical trial design with ATR kinase inhibitors.

## Additional files

Additional file 1: Figure S1. Lung cancer cell lines were radiosensitized by ATM kinase inhibitor. Cells were prepared in suspension and treated with KU60019 and increasing doses of IR. Cells were seeded in 60 mm petri dishes. Drug treatments were removed 17 h post-IR. After 10 days, colonies were stained with crystal violet stain. A representative example of three experiments is shown. (PDF 555 kb)
Additional file 2: Table S1. Gemcitabine, CHK1i and ATRi IC50 values and combination indices (CI-ED50) in human lung cancer cell lines (mean, SD). **Table S2.** Gemcitabine IC50 values and potentiation by ATMi in human lung cancer cell lines (mean, SD). **Table S3.** Carboplatin, CHK1i and ATRi IC50 values and combination indices (CI-ED50) in human lung cancer cell lines (mean, SD). (DOC 47 kb)
Additional file 3: Figure S1. Alterations in 15 FA genes were identified in 212 lung squamous cell carcinomas (TCGA). Amplification, homozygous deletion, up-regulation RNA, down-regulation RNA and mutation are shown. (PDF 1919 kb)

## Abbreviations

aCGH: array-comparative genomic hybridization
AT: Ataxia telangiectasia
ATCC: American type culture collection
ATM: Ataxia telangiectasia mutated
ATR: ATM and Rad3-related
DMSO: Dimethyl sulfoxide
DSB: DNA double-strand break
FA: Fanconi anemia
HRR: Homologous recombination repair
ICL: Interstrand crosslink
IR: Ionizing radiation
MMC: Mitomycin C
NSCLC: Non-small-cell lung carcinoma
SDS-PAGE: Sodium dodecyl sulfate polyacrylamide gel electrophoresis
TCGA: The cancer genome atlas
