# Supplementary figures and images for: Functional analyses of ATM, ATR and Fanconi anemia proteins in lung carcinoma: ATM, ATR and FA in lung carcinoma
Source: BMC Cancer. 2015 Oct 5;15:649. doi: 10.1186/s12885-015-1649-3 (PMC4595318; doi:10.1186/s12885-015-1649-3)

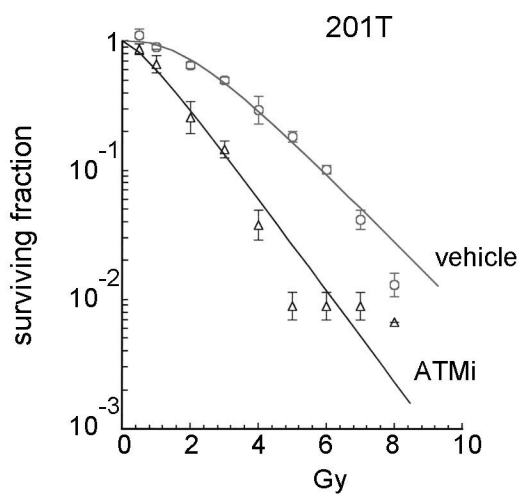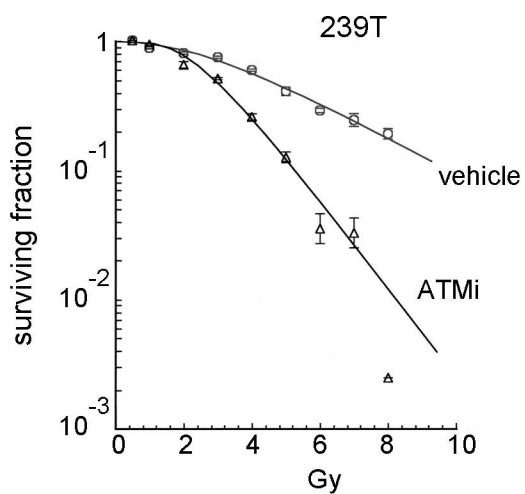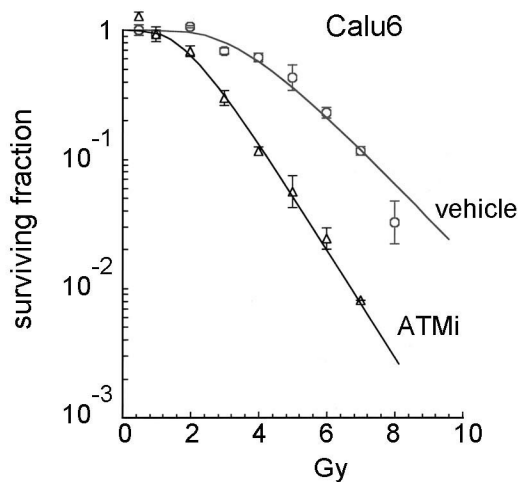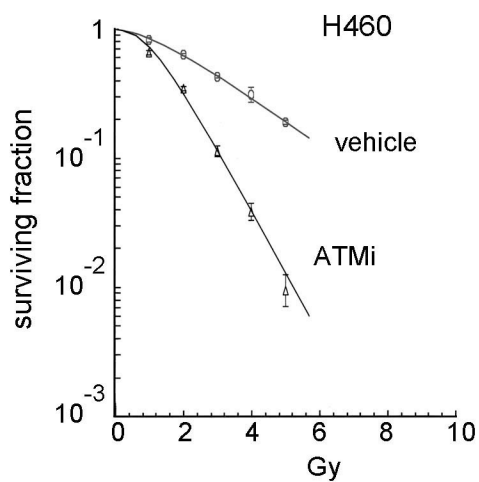

Supplement: Additional file 1: Figure S1. — Lung cancer cell lines were radiosensitized by ATM kinase inhibitor. Cells were prepared in suspension and treated with KU60019 and increasing doses of IR. Cells were seeded in 60 mm petri dishes. Drug treatments were removed 17 h post-IR. After 10 days, colonies were stained with crystal violet stain. A representative example of three experiments is shown. (PDF 555 kb) [file 12885_2015_1649_MOESM1_ESM.pdf]

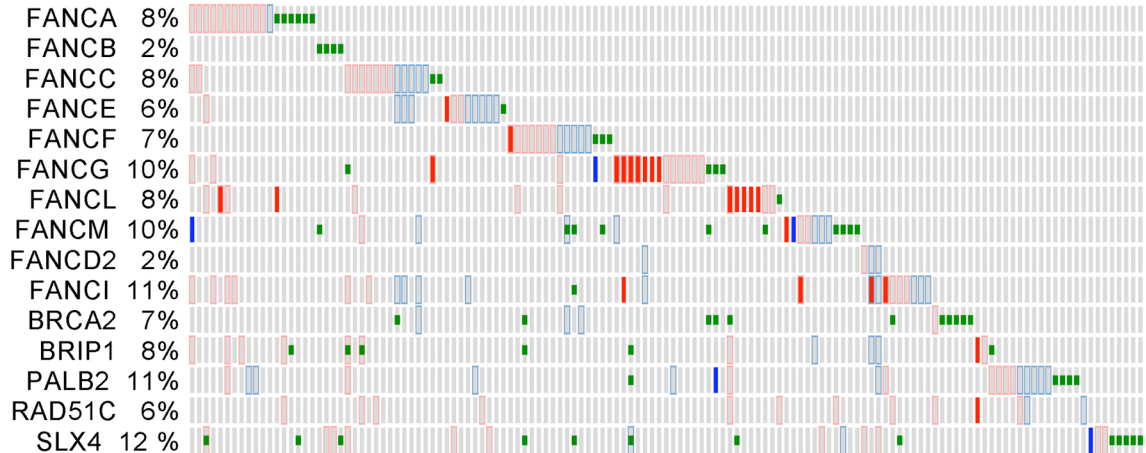

Amplification  
 Homozygous deletion  
 Up-regulation (RNA)  
 Down-regulation (RNA)  
 Mutation

Supplement: Additional file 3: Figure S1. — Alterations in 15 FA genes were identified in 212 lung squamous cell carcinomas (TCGA). Amplification, homozygous deletion, up-regulation RNA, down-regulation RNA and mutation are shown. (PDF 1919 kb) [file 12885_2015_1649_MOESM3_ESM.pdf]
