# Supplementary material for: Functional analyses of ATM, ATR and Fanconi anemia proteins in lung carcinoma: ATM, ATR and FA in lung carcinoma
Source: BMC Cancer. 2015 Oct 5;15:649. doi: 10.1186/s12885-015-1649-3 (PMC4595318; doi:10.1186/s12885-015-1649-3)
Supplement: Additional file 2: Table S1. — Gemcitabine, CHK1i and ATRi IC50 values and combination indices (CI-ED50) in human lung cancer cell lines (mean, SD). Table S2. Gemcitabine IC50 values and potentiation by ATMi in human lung cancer cell lines (mean, SD). Table S3. Carboplatin, CHK1i and ATRi IC50 values and combination indices (CI-ED50) in human lung cancer cell lines (mean, SD). (DOC 47 kb) [file 12885_2015_1649_MOESM2_ESM.doc]

**SUPPLEMENTAL TABLES**

**Table 1. Gemcitabine, CHK1i and ATRi IC_50_ values and combination indices (CI-ED_50_) in human lung cancer cell lines (mean, SD).**

|  | **Gemcitabine** | **CHK1i** | **ATRi** | **Gemcitabine-ATRi** | **Gemcitabine-CHK1i** |
| --- | --- | --- | --- | --- | --- |
| **Cell line** | **IC_50_**  **(ng/mL)** | **IC_50_**  **(ng/mL)** | **IC_50_**  **(ng/mL)** | **CI-ED_50_** | **CI-ED_50_** |
| **54T** | **200 (143)** | **67.8 (31.1)** | **1040 (676)** | **1.16 (0.82)** | **0.31 (0.16)** |
| **201T** | **7.87 (1.95)** | **30.8 (10.3)** | **432 (99)** | **1.08 (0.50)** | **0.98 (0.75)** |
| **239T** | **6.45 (1.02)** | **42.7 (24.5)** | **573 (386)** | **0.54 (0.16)** | **0.73 (0.11)** |
| **Calu6** | **6.05 (1.45)** | **93.4 (52.8)** | **708 (454)** | **0.78 (0.31)** | **0.48 (0.20)** |
| **H460** | **3.59 (1.56)** | **44.4 (24.1)** | **350 (152)** | **1.11 (0.12)** | **0.89 (0.41)** |

**Table 2. Gemcitabine IC_50_ values and potentiation by ATMi in human lung cancer cell lines (mean, SD)**

|  | **Gemcitabine** | **Gemcitabine+ATMi** | **Potentiation factor** |
| --- | --- | --- | --- |
| **Cell line** | **IC_50_ (ng/mL)** | **IC_50_ (ng/mL)** | **IC_50_-ratio** |
| **54T** | **63.6 (27.6)** | **69.3 (34.6)** | **0.94 (0.09)** |
| **201T** | **9.23 (0.19)** | **17.6 (5.7)** | **0.55 (0.15)** |
| **239T** | **3.29 (1.09)** | **2.49 (1.03)** | **1.67 (1.35)** |
| **Calu6** | **2.68 (1.18)** | **1.48 (1.20)** | **2.34 (1.17)** |
| **H460** | **5.18 (1.29)** | **2.39 (0.29)** | **2.16 (0.46)** |

**Table 3. Carboplatin, CHK1i and ATRi IC_50_ values and combination indices (CI-ED_50_) in human lung cancer cell lines (mean, SD).**

|  | **Carboplatin** | **CHK1i** | **ATRi** | **Carboplatin-ATRi** | **Carboplatin-CHK1i** |
| --- | --- | --- | --- | --- | --- |
| **Cell line** | **IC_50_**  **(µg/mL)** | **IC_50_**  **(ng/mL)** | **IC_50_**  **(ng/mL)** | **CI-ED_50_** | **CI-ED_50_** |
| **54T** | **141 (48)** | **105 (72)** | **1057 (351)** | **1.70 (1.68)** | **0.26 (0.08)** |
| **201T** | **177 (84)** | **145 (107)** | **520 (190)** | **0.59 (0.23)** | **0.48 (0.45)** |
| **239T** | **49.5 (19.0)** | **69.0 (34.2)** | **443 (153)** | **0.77 (0.32)** | **0.42 (0.06)** |
| **Calu6** | **40.5 (1.9)** | **82.9 (35.1)** | **312 (93)** | **1.07 (0.79)** | **0.66 (0.26)** |
| **H460** | **40.7 (5.0)** | **82.0 (21.9)** | **346 (82)** | **0.77 (0.34)** | **0.35 (0.06)** |
